# Emerging Therapeutic Strategies for Limbal Stem Cell Deficiency

**DOI:** 10.1155/2018/7894647

**Published:** 2018-06-27

**Authors:** Ying Dong, Han Peng, Robert M. Lavker

**Affiliations:** ^1^Department of Ophthalmology, The First Affiliated Hospital, Chinese PLA General Hospital, Beijing 100048, China; ^2^Department of Dermatology, Northwestern University, Chicago, IL 60611, USA

## Abstract

Identification and characterization of the limbal epithelial stem cells (LESCs) has proven to be a major accomplishment in anterior ocular surface biology. These cells have been shown to be a subpopulation of limbal epithelial basal cells, which serve as the progenitor population of the corneal epithelium. LESCs have been demonstrated to play an important role in maintaining corneal epithelium homeostasis. Many ocular surface diseases, including intrinsic (e.g., Sjogren's syndrome) or extrinsic (e.g., alkali or thermal burns) insults, which impair LESCs, can lead to limbal stem cell deficiency (LSCD). LSCD is characterized by an overgrowth of conjunctival-derived epithelial cells, corneal neovascularization, and chronic inflammation, eventually leading to blindness. Treatment of LSCD has been challenging, especially in bilateral total LSCD. Recently, advances in LESC research have led to novel therapeutic approaches for treating LSCD, such as transplantation of the cultured limbal epithelium. These novel therapeutic approaches have demonstrated efficacy for ocular surface reconstruction and restoration of vision in patients with LSCD. However, they all have their own limitations. Here, we describe the current status of LSCD treatment and discuss the advantages and disadvantages of the available therapeutic modalities.

## 1. Introduction

The functions of cornea include protecting the delicate internal parts of the eye and allowing proper transmission of light. The corneal epithelium is the outermost layer of cornea, which is a crucial barrier against mechanical, chemical, and pathogenic insults. In fulfilling its barrier function, this self-renewing stratified epithelium turns over every 5–7 days. The self-renewal of the corneal epithelium is governed by the stem cells that reside in the basal layer of the limbal epithelium, adjacent to the corneal epithelium [1]. The first observation that the limbal epithelium might be involved in replenishing the corneal epithelium came from Davanger and Evensen who noted “streaking” of the pigmented limbal epithelium into the corneal epithelium following an insult [2]; however, they did not suggest the involvement of stem cells in this process. In 1986, Schermer et al. proposed that the corneal epithelial stem cells resided in the limbal epithelial basal cells [3]. It was the landmark paper. In 1989, Cotsarelis et al. for the first time proved this hypothesis by demonstrating that label-retaining cells (a marker of slow-cycling cells, which is a characteristic of stem cells) were preferentially located in the basal layer of the limbal epithelium and not in the corneal epithelium [1]. Since then, the biology of limbal epithelial stem cells (LESCs) has attracted many attentions.

## 2. Characteristics of Limbal Epithelial Stem Cells

LESCs are morphologically small, have a high nuclear-to-cytoplasm ratio, and are relatively undifferentiated cells with rare cycling and high proliferative capacity [4, 5]. The difference of the limbal epithelial stem cells and corneal epithelium is shown in Table 1. More importantly, LESCs have the capability to regenerate the entire corneal epithelium [6]. Similar to other somatic stem cells, LESCs highly express stem cell markers, including transporters (e.g., ABCG2 and ABCB5) [7, 8], transcription factors (e.g., C/EBPδ, Bmi-1, ΔNp63α, and Pax6) [9–11], cell adhesion molecules and receptors (e.g., N-cadherin, integrins α9 and β1, and Frizzled (Fz)7), and cytokeratins (e.g., CK15, CK14, and CK19) [12–14] [15].

### 2.1. Low Differentiation

Limbal epithelial basal cells are relatively undifferentiated and thus lack the expression of differentiation markers such as keratin 3, keratin 12 [16], and connexin 43, which is associated with a more differentiated cell [17].

### 2.2. Infrequent Cycling

Stem cells are commonly believed to cycle infrequently [18]. This characteristic has been postulated to enable stem cells to preserve their proliferative capacity and to minimize DNA replication-associated errors [19, 20]. Utilizing this characteristic of infrequent cycling, LESCs were identified using the “label-retaining cells” (LRCs) technique. First, all of the dividing cells (including stem cells) are labeled by continuous exposure to either tritiated thymidine (^3^H-Tdr) or bromodeoxyuridine (BrdU). After a chasing period (usually 4–8 weeks), the labeling signal in the rapidly dividing TA cells is diminished due to dilution or by transiting out of the tissue due to differentiation, whereas the slow-cycling stem cells still retain their labeling. Application of this labeling technique to mouse limbal/corneal epithelia revealed that the LRCs were exclusively localized in the basal layer of the limbal epithelium. In contrast, the peripheral and central corneal epithelia contained no LRC, which was compelling evidence that the corneal epithelial stem cells were located in the limbal epithelium [1, 19].

### 2.3. High Capacity for Self-Renewal and Proliferation

LESCs have high proliferative capacity, which is demonstrated in vitro by an ability to generate holoclone colonies [21]. On the contrary, in the transit-amplifying (TA) cells, only the progeny of LESCs are able to produce meroclone and paraclone colonies [21]. Holoclone, meroclone, and paraclone colonies represent three different proliferative capacity clonogenicity. Holoclone colonies are believed to be derived from stem cells and have the greatest proliferative capacity. Meroclone colonies are believed to be derived primarily from TA cells and have less cellular division potential. Finally, paraclone colonies are thought to be derived from mature TA cells and have the least proliferative potential. Cells from the stem cell-enriched limbal epithelium can undergo 80 to 100 cell division cycles and are capable to form holoclone colonies, whereas cells from the central corneal epithelium undergo 15 cell divisions maximally and only form paraclone colonies [21].

### 2.4. Limbal Niche

The limbal stem cell niche is a specific and highly regulated microenvironment, which is required for harboring and maintaining LESCs [22–26]. It has been suggested that the human limbal stem cell niche is located in the palisades of Vogt (recently termed “crypts”) [27–30]. Limbal epithelial crypts (LECs) have been demonstrated to extend from the peripheral aspects of an interpalisade rete ridge and further into the conjunctival stroma as a solid chord of cells measuring up to 120 *µ*m [27]. It is generally believed that the niche consists of three components: (i) cell-cell interactions between stem cells and TA cells, (ii) the basement membrane, and (iii) the extracellular matrix and mesenchymal cells directly adjacent to and beneath the basement membrane. Disruption of the limbal niche by various pathological conditions (e.g., severe immune response and wounding) can lead to LSCD.

## 3. Limbal Stem Cell Deficiency

Clinically, LSCD is caused by the depletion or dysfunction of LESCs, which leads to the inability to sustain corneal epithelial homeostasis [31–35]. Patients often present with pain, photophobia, and decreased vision in the acute stages of LSCD. Biomicroscopy shows conjunctival hyperemia, loss of the palisades of Vogt, and a “whorled-like” corneal epithelium [36, 37]. LSCD is also associated with poor epithelial adhesion, resulting in recurrent erosions and persistent corneal epithelial defects. At the chronic stage, the ocular surface is scarred and extensively neovascularized.

## 4. Clinical Treatments of LSCD

Clinical treatment of the LSCD varies based on the severity and extent of involvement. For those patients with mild and moderate LSCD, treatments involve the control of the symptoms and causes. For patients with severe LSCD, it is necessary to undergo ocular surface reconstruction (OSR). OSR is a series of procedures to reconstitute the anatomic and physiologic ocular surface, including amniotic membrane transplantation (AMT), conjunctival limbal grafting, simple limbal epithelial transplantation (SLET), and cultivated limbal epithelial transplantation (CLET) [33, 38–41]. The recent progress in understanding limbal epithelial stem cell biology has formed foundations for novel cell-based therapeutic strategies.

### 4.1. Amniotic Membrane Transplantation

Amniotic membrane transplantation (AMT) is a method to help recreate the integrity of the ocular surface. The amniotic membrane (AM) consists of an overlying basement membrane with a rich extracellular matrix, including heparin sulfate proteoglycans, laminin, and collagens. These components act as a scaffold for the epithelial cells [38, 42, 43]. The AM also contains various growth factors, protease inhibitors, and anti-inflammatory and antiangiogenic factors and thus exerts potent anti-inflammatory and antiscarring effects [44]. The AM mimics the natural stem cell niche and therefore has the potential to enhance the self-renewal of limbal epithelial stem cells [45]. For the past decade, the amniotic membrane has become an ideal substrate for various transplantation procedures on the ocular surface [46, 47].

### 4.2. Autologous Conjunctival Limbal Transplant

Traditional autologous limbal transplantation has a long history. In 1989, Kenyon and Tseng described a large series (26 consecutive cases) of conjunctival limbal autograft (CLAU) in patients with unilateral ocular surface diseases [48]. A six-month follow-up study showed that the CLAU resulted in the improvement in visual acuity, rapid surface healing, and stable epithelial adhesion without recurrent erosion or persistent epithelial defect, as well as a regression of corneal neovascularization. This pioneer work identified that the transplanted limbal tissue can rehabilitate the corneal surface [48]. However, traditional autologous limbal transplantation requires a large limbal epithelial biopsy from a healthy eye, which increased the potential of damaging the donor eye [49].

### 4.3. Allograft Limbal Stem Cell Transplant

For patients with a total bilateral LSCD, allograft limbal stem cell transplant is one of the approaches to reconstruct the ocular surface [6]. The conjunctiva and limbus, presumably including stem cells, can come from living relative (parent or sibling) or cadaveric limbal tissues. Allograft limbal stem cell transplant can provide immediate postoperative epithelialization and rapidly reconstruct the ocular surface. However, to avoid rejection of the allograft, systemic immunosuppression is required. Adverse effects related to long-term immunosuppression are common, including anemia, hyperglycemia, elevated creatinine, and elevated levels of liver function markers [50, 51]. Interestingly, a long-term study showed that eventually, only recipient DNAs were detectable in the regenerated epithelium of the majority of the successful cases. This suggests that the allografted limbal epithelium promotes regeneration of the corneal epithelium in patients with LSCD, at least in part, by activating residual stem cells and enhancing their self-renewal [52]. It is possible that allografted limbal stem cells secrete factors that are necessary for maintaining stem cell homeostasis. It is very important to elucidate what these factors are and whether direct application of such factors onto the ocular surface can restore the corneal epithelium.

### 4.4. Simple Limbal Epithelial Transplantation (SLET)

In 2012, Sangwan et al. introduced a simple limbal epithelial transplantation (SLET) [53]. In this technique, a fresh amniotic membrane had been attached on the cornea by a fibrin glue; a small (2 × 2 mm) donor limbal graft from the unaffected eye was harvested and divided into tiny pieces and then seeded on the AM. This technique provides a simple approach that makes the LESCs expand in vivo [54–60].

A multicenter study on 68 eyes from patients who underwent SLET for unilateral LSCD reported promising results [61]. Clinical success was achieved in 57 (84%) cases. With a median follow-up of 12 months, the survival probability exceeded 80%. Recently, long-term clinical outcomes of a large cohort of patients (125 cases) with unilateral LSCD occurring after ocular burns showed that 76% patients maintained a successful outcome. In addition to surface restoration, most patients undergoing SLET reported a significant improvement in visual acuity. Immunohistochemistry revealed successful regeneration of the normal corneal epithelium (CK3(+)/12(+)) without admixture of conjunctival cells (Muc5AC(−)/CK19(−)) and replenishment of the limbal stem cell (ΔNp63α(+)/ABCG2(+)) reserve [62]. The SLET has a similar success rate to the traditional autologous limbal transplantation. Better yet, different from conjunctival limbal grafting, autologous SLET requires only a tiny limbal tissue from the unaffected eye carrying minimal risk to the donor eye. Additionally, in comparison with ex vivo cultivated limbal epithelial transplantation, SLET does not need clinical-grade laboratory support, which has the advantage of low cost and is easily replicable by practicing corneal surgeons [63].

## 5. Cell-Based Therapy

Cell therapy involves tissue engineering techniques and the idea of stem cell plasticity for achieving corneal epithelial regeneration. This approach represents new potential therapeutic modalities. The underlying principal is to use the least amount of tissue to ex vivo expand cells into an epithelial sheet on carriers and to reconstruct severely damaged ocular surfaces.

### 5.1. Cultivated Limbal Epithelial Transplantation (CLET)

Transplantation of autologous cultures of limbal epithelial stem cells was first reported by Pellegrini et al. [64]. Two patients with unilateral LSCD at the severe chronic stage of alkali burns received CLET. Limbal epithelial cells from a 1-2 mm^2^ limbal biopsy sample were expanded in vitro on a feeder layer consisting of nonproliferating 3T3-J2 feeder cells and a polymerized fibrin matrix. Confluent cultured limbal epithelial sheet was placed on a corneal wound bed. Two-year follow-up showed that the regenerated corneal epithelium was stable. In 2010, Pellegrini et al. reported a long-term clinical investigation of CLET with the large samples (112 LSCD patients) [65]. In this study, 76.6% eyes showed permanent restoration of a transparent, renewing the corneal epithelium. This suggests that CLET is an effective method to reconstruct the ocular surface [66, 67]. Interestingly, the failure of transplantation of the limbal epithelial cultures is significantly associated with the lack of holoclone-forming cells (stem cells) in limbal epithelial cultures. Therefore, it is of clinical significance to identify regulators that could be pharmacologically targeted to enhance the stem cell number.

### 5.2. New Approaches to Maximize Ex Vivo Expansion of Limbal Epithelial Cells

A major challenge for CLET-based therapies is to maintain LESC homeostasis and enhance the self-renewal of LESCs in limbal epithelial cultures during ex vivo expansion. MicroRNAs (miRNAs) are emerging as important controllers of stem cell potency, proliferation, and differentiation [68–74]. For example, miR-205 plays a potentially important role in regulating cell proliferation and survival, via targeting the PI3K/Akt pathway [75, 76]. Such a regulation could impact effective expansion of limbal epithelial cells. Another critical miRNA family is miR-103/107 that is preferentially expressed in the stem cell-enriched limbal epithelium and targets novel proteins involved in processes related to stem cell behavior [77]. miR-103/107 targets p90RSK2, a kinase that regulates G0/G1 progression, and this helps to maintain a slow-cycling phenotype [78]. miRs-103/107 also promote increased holoclone colony formation by regulating MAP3K7 signaling and JNK activation through noncanonical Wnt signaling. By targeting NEDD9 (HEF1), miR-103/107 ensures maintenance of the essential stem cell niche molecule, E-cadherin (E-cad) in limbal keratinocytes [78]. By targeting protein tyrosine phosphatase, receptor type M (PTPRM), miR-103/107 maintains low levels of Cx43, which is a feature of several stem cell-enriched epithelia [78]. Collectively, miR-103/107 plays critical roles in the regulation of stem cell proliferation and the interaction of stem cells with their surrounding cells [79]. These findings form a foundation for development of a novel approach to improve the preservation of limbal stem cells in ex vivo cultures prior to CLET. It has been demonstrated that microRNAs can be topically delivered into limbal/corneal epithelia [78]. Thus, it has a clinical potential to topically administer miR-103/107 into limbal/corneal epithelia, which may activate and preserve the remaining limbal stem cells of patients with LSCD.

It is well established that autophagy is required for stem cell homeostasis in various tissues [80]. Consistent with this idea, we have demonstrated that the autophagy activity is significantly higher in the basal layer of the limbal epithelium compared with the corneal epithelium. More interestingly, the holoclone colony-forming ability was markedly diminished in limbal epithelial cells when autophagy was blocked [81]. These new findings suggest that autophagy is a positive process for maintaining stem cells [82]. The signaling pathways that regulate autophagy specifically in the limbal basal layer need to be elucidated.

### 5.3. Cultivated Oral Mucosal Epithelial Transplantation (COMET)

Bilateral LSCD patients who have no remaining LESCs can turn to autologous cultivated oral mucosal epithelial transplantation (COMET). It has been shown that COMET is a feasible substitute for allogenic limbal stem cell transplantation without the need for long-term systemic immunosuppression [83–89].

The cultivated oral epithelial cells formed a stratified tissue. The tissue-engineered oral epithelium expressed proliferation and progenitor markers Ki-67 and p63 in the basal layer of the cell sheets, suggesting that the epithelium had regenerative capacity [90]. The transplanted epithelium also expressed K3, K19, Ki-67, p63, p75, and the cornea-specific PAX6 and K12 [90]. This study confirms that the oral cells, transplanted to the corneal surface, can survive and stably reconstruct the ocular surface. They acquire some of the corneal epithelial-like characters at the ectopic site. However, compared with cultured limbal epithelial cells, COMET has significantly higher angiogenic potential. In addition, the underlying mechanisms involved in the transformation of the oral mucosal epithelial cells into the differentiated corneal epithelium remains unclear.

### 5.4. Mesenchymal Stem Cells

Mesenchymal stem cells (MSCs) are a group of fibroblast-like multipotent mesenchymal stromal cells [91]. MSC can be isolated from a wide range of tissues, including bone marrow, umbilical cord, adipose tissue [92], and corneal stroma [93]. Because of an urgent need for alternative autologous stem cell sources for bilateral LSCD, MSC has been tested in the treatment of LSCD. Holan et al. demonstrated that bone marrow MSCs (BM-MSCs) had similar therapeutic effects in the experimental LSCD model of alkali-injured rabbit eyes compared with LESCs [94]. Some studies suggested the differentiation of MSCs into corneal epithelial cells. However, the precise mechanism by which MSCs differentiate into corneal epithelial cells remains elusive. It has also been suggested that MSCs produce growth factors that can support the growth of residual corneal epithelial cells and LESCs [95]. Recent researches by Shaharuddin et al. found that cultured limbal MSCs expressed the common putative limbal stem cell markers, which demonstrated limbal-derived MSC-exhibited plasticity [96].

## 6. Conclusion

Basic science has contributed greatly to our understanding of the location, function, regulation of proliferation, and differentiation of limbal epithelial stem cells. Conventional autogenic and allogenic conjunctival limbal transplantation is an effective method but is limited by tissue availability. To overcome the shortage of donor-based tissues, scientists now focus their attention on cell-based therapy. With the refinement of in vitro culture and expansion techniques, and improved scaffolds and matrices, it is anticipated that a new generation of regenerative procedures will be available for use in the clinic to ultimately resolve the problem of LSCD. Finally, an emerging idea that supplies factors in vivo to activate and preserve the remaining limbal stem cells may lead to a pharmacological therapy which will ultimately replace surgery for treatment of corneal diseases with limbal stem cell deficiency.

## Figures and Tables

**Table 1:** The features of corneal epithelial cells and limbal epithelial stem cells.

	Limbal epithelial stem cells (LESCs)	Corneal epithelium (CE)
Morphology	High nucleus-to-cytoplasm ratio; smaller than CE (10.1 ± 0.8 *µ*m)	Lower nucleus-to-cytoplasm ratio; column cell (17.1 ± 0.8 *µ*m)
Blood supply	High vascularization	Avascular
Clonogenicity	Holoclones	Paraclones
Pigmentation	Intrinsic melanogenesis	Absent pigment, transparency
Epithelial cell marker	K5 and K14	K3, K12, and Cx43
Putative stem cell marker	ABCG2, K19, vimentin, integrin α9 and so on	—
Metabolic activity	Low	High
Cell cycling	Slow cycling	Fast cycling
